# A systematic review and meta-analysis of the relationship between body size and testicular cancer

**DOI:** 10.1038/sj.bjc.6605934

**Published:** 2010-10-26

**Authors:** C C Lerro, K A McGlynn, M B Cook

**Affiliations:** 1 Yale School of Public Health, Yale University, New Haven, CT, USA; 2 Hormonal and Reproductive Epidemiology Branch, Division of Cancer Epidemiology and Genetics, NCI, NIH, DHHS, Bethesda, MD, USA

**Keywords:** body height, body mass index, body weight, meta-analysis, review, systematic, testicular neoplasms

## Abstract

**Background:**

Studies assessing the relationships of anthropometry and testicular germ-cell tumour (TGCT) have reported heterogeneous findings.

**Methods:**

We undertook a systematic review and meta-analysis of the associations between adult height, weight, body mass index (BMI), and testicular cancer. Search strategies were conducted in PubMed, EMBASE, Scopus, and Web of Science on 26 May 2009. Studies that met our inclusion criteria were included in meta-analytic models using STATA 11.

**Results:**

A total of 3255 references were retrieved, of which 14 met the inclusion criteria. Random effects meta-analysis found adult height (odds ratio (OR) per 5-cm increase 1.13, 95% confidence interval (CI) 1.07–1.19, *P*<0.001) and weight (OR overweight *vs* normal 0.92, 95% CI 0.86–0.98, *P*=0.011) to be associated with TGCT. The meta-analysis of weight and TGCT produced a summary estimate, which indicated no association, although an analysis restricted studies to North American was suggestive of association (OR per 1-kg increase 1.01, 95% CI 1.00–1.01, *P*<0.001).

**Conclusions:**

This systematic review and meta-analysis has found evidence for a positive association of adult height and TGCT, and tentative evidence for an inverse association of BMI and TGCT.

Testicular cancer is a relatively rare malignancy in the general population, but is the most commonly occurring cancer among young men in many countries (Bjorge *et al*, 2006; Bray *et al*, 2006; Shah *et al*, 2007). Established risk factors for testicular cancer include European ancestry (Newell *et al*, 1987), cryptorchidism (Garner *et al*, 2005), family history of testicular cancer, and previous diagnosis of testicular cancer (McGlynn, 2001). In addition, meta-analytic synthesis of all available pre- and perinatal data has implicated low-birth order, maternal bleeding during pregnancy, larger sibship size, low-birth weight, twinning, gestational age, and inguinal hernia in the etiopathogenesis of testicular cancer (Cook *et al*, 2009, 2010). There is also an increasing amount of evidence that height and potentially overall body size, variables which are largely determined during early development, may also be related to risk, although the literature is not entirely consistent (Dieckmann *et al*, 2009).

Adult height is determined in early life by a combination of genetics, health and nutrition (Albanes *et al*, 1988). There is some evidence that high fat or high dairy intake in childhood is associated with both adult body size and risk for testicular cancer (Davies *et al*, 1996; Stang *et al*, 2006; McGlynn *et al*, 2007; Lagorio *et al*, 2008). Growth factors and growth factor binding proteins have also been examined as possible risk factors for testicular cancer (Chia *et al*, 2008). Although it is unlikely that height, weight or body mass index (BMI) are direct determinants of testicular cancer risk, it is probable that some underlying factor influences both body size and risk of testicular cancer. To clarify these relationships and to investigate inconsistencies in the literature, we undertook a systematic review and meta-analysis of adult height, weight, and BMI in relation to the risk of testicular cancer.

## Materials and methods

With assistance from the National Institutes of Health Library, a comprehensive search strategy was designed to incorporate all possible terms and keywords relating to testicular cancer and body size. A search of the literature was executed on 26 May 2009 using four major scientific literature databases, PubMed (National Centre for Biotechnology Information, US National Library of Medicine, USA; 1900–2009), EMBASE (Elsevier BV, The Netherlands; 1974–2009), Scopus (Elsevier BV, The Netherlands; 1823–2009), and Web of Science (Thomson Reuters, USA; 1900–2009). Search strategies are available from the authors on request. Titles, abstracts, and keywords were assessed by two reviewers (CCL and MBC). Articles were compiled and managed using Endnote X3 software (Thomson Reuters, 2009).

To be included in the meta-analysis, studies had to meet the following criteria; (1) have at least 20 cases and 20 controls in the analysis; (2) recruited only incident cases to avoid survivorship bias; and (3) have a valid control group (no case-series or case-study designs were included). For the meta-analysis of adult weight, it was required to have the weight ascertained shortly before or around the time of diagnosis. For the meta-analysis of adult height, it was required to have the height ascertained at age 18 years or older. Both of these conditions had to be met for a study to be eligible for the meta-analysis of BMI. No restrictions were placed on year or language of publication. If a single study had reported results in more than one publication, that with the largest number of cases was included.

Variables abstracted from studies included country of study population, institute or hospital, years of study, study design, patient selection criteria and source, mean age of study participants, mean age at diagnosis, race/ethnicity, data ascertainment method, and whether the study included all testicular tumours or only testicular germ cell tumours (TGST) (the most common type of testicular tumours).

### Statistical analyses

All analyses were performed in STATA v.11 (StataCorp, 2009). Meta-analyses of the relevant continuous variables (height, weight, BMI) were performed to estimate odds of testicular cancer per unit increase, according to methods previously outlined by Chene and Thompson (1996). BMI (kg m^−2^) was also examined as an ordinal variable using groupings of normal (BMI <25), overweight (BMI 25–29.9), and obese (BMI ⩾30). A random-effects meta-analysis was used according to the methods of DerSimonian and Laird (1986). The *I*^*2*^ value and its 95% uncertainty intervals (UIs) were used to assess the consistency of the study-specific estimates (Higgins *et al*, 2003). A funnel plot of the log odds ratio (OR) against the s.e. of the log OR was examined as an indicator of publication bias. Egger and Begg's tests were performed to test for small-study effects (Begg and Mazumdar, 1994; Egger *et al*, 1997). A sensitivity analysis was conducted whereby each study was omitted in turn and the summary estimate recalculated to determine the influence of each study.

Meta-regression was performed for each of the body-size variables under consideration to investigate heterogeneity (Higgins and Thompson, 2004). Variables for meta-regression were designated *a priori* and included year of study, country, study design, ascertainment method, institute or hospital, time of study, time of follow-up, and whether the study included all testicular tumours or only germ cell tumours. The results of a meta-regression are only reported and discussed if the *P*-value was less than the arbitrary value of 0.05.

## Results

From our comprehensive and sensitive search strategy we collated 3255 articles of potential interest. Two authors (CL and MBC) reviewed titles and abstracts of these articles and identified 189 articles of potential interest (101 original research articles and 88 review articles). The full-text of each article was retrieved and the manuscript was considered against the stipulated selection criteria for our review. Four additional original research articles were identified through review of citations of the full-text articles, for a total of 195 relevant articles. Any discrepancies regarding inclusion were discussed among all the three authors until consensus was reached. In total, 21 authors were contacted and requested for further information, of whom 19 replied and 8 provided additional information or unpublished data. In total, 14 articles met the selection criteria, provided the appropriate information or data to calculate OR, and were able to be included in at least two of the three (height, weight, and BMI) meta-analyses (Table 1). We did not exclude articles based on type of testicular cancer (germ cell tumours or all tumours), but all the articles matching our inclusion criteria studied only TGCT.

Adult height and weight were used to calculate BMI of individuals of each study. Included studies used weight at TGCT diagnosis (Petridou *et al*, 1997; Hardell *et al*, 2006; Dieckmann *et al*, 2009) or weight approximately 1 year before diagnosis (Davies *et al*, 1990; UKTCSG, 1994; Walcott *et al*, 2002; Stang *et al*, 2006; McGlynn *et al*, 2007), 2 or more years before diagnosis (Richiardi *et al*, 2003; Pan *et al*, 2004), at age 20 (Swerdlow *et al*, 1989; Dieckmann and Pichlmeier, 2002), at age 21 (Gallagher *et al*, 1995), or at some unspecified time before diagnosis (Bjorge *et al*, 2006).

**Figure 1:**
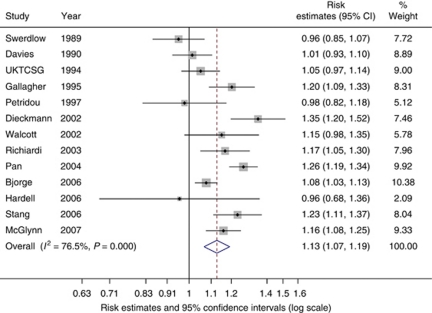
Forest plot of the association between height (per 5 cm) and testicular cancer risk. Each study-specific estimate is represented by a small solid diamond with adjoining horizontal lines, which represent the 95% confidence intervals. The size of the grey square surrounding the study-specific estimates represents the weight of each study in the meta-analysis. The diamond with an ascending dashed line from its upper point is the summary estimate. The width of diamond represents the 95% confidence intervals of the summary estimate.

**Figure 2:**
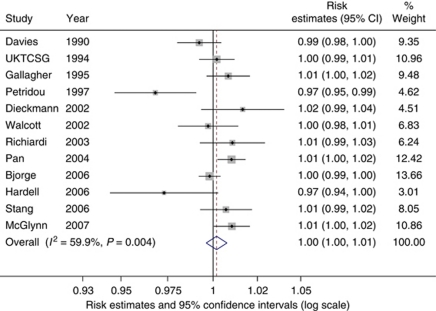
Forest plot of the association between weight (per kg) and testicular cancer risk. Each study-specific estimate is represented by a small solid diamond with adjoining horizontal lines which represent the 95% confidence intervals. The size of the grey square surrounding the study-specific estimates represents the weight of each study in the meta-analysis. The diamond with an ascending dashed line from its upper point is the summary estimate. The width of diamond represents the 95% confidence intervals of the summary estimate.

In the meta-analysis of the association between adult height and TGCT, 13 studies were included which, in combination, produced a summary OR of 1.13 per 5 cm increase in height (95% CI: 1.07–1.19, *P*<0.001; Figure 1). The heterogeneity between studies, as measured by the *I*^*2*^ value, was 77% (95% UI 60–86%). The high heterogeneity was not explained by meta-regression of those variables specified *a priori*. The Egger and Begg's tests both indicated no small-study effects (*P*=0.819 and *P*=0.714, respectively). A total of 12 studies contributed to the analysis of the association between adult weight and TGCT (Figure 2). Findings indicated that adult weight was unrelated to TGCT, with a summary risk estimate of 1.00 per 1 kg increase in weight (95% CI: 1.00–1.01, *P*=0.508). The *I*^*2*^ value measuring between-study heterogeneity was 60% (95% UI: 24–79%). Egger and Begg's tests found no evidence for any small-study effects (*P*=0.480 and *P*=0.337, respectively).

**Figure 3:**
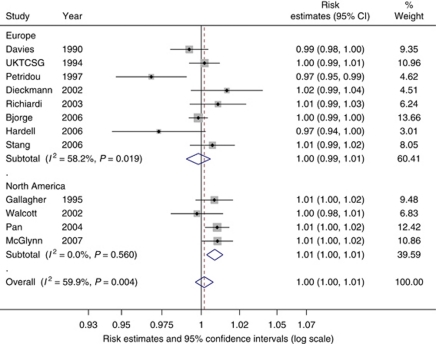
Forest plot of the association between weight (per kg) and testicular cancer risk stratified by continent. Each study-specific estimate is represented by a small solid diamond with adjoining horizontal lines which represent the 95% confidence intervals. The size of the grey square surrounding the study-specific estimates represents the weight of each study in the meta-analysis. The diamond with an ascending dashed line from its upper point is the summary estimate. The width of diamond represents the 95% confidence intervals of the summary estimate.

Meta-regression of adult weight by continent (North America against Europe) was of borderline statistical significance (*P*=0.058). A stratified forest plot graphically illustrates the differences in the relationship between adult weight and TGCT by continent (Figure 3). The European studies had a summary risk estimate of 1.00 per 1 kg increase in weight (95% CI: 0.99–1.01, *P*=0.643) with an *I*^*2*^ value for between-study heterogeneity of 58%, whereas North-American studies produced a borderline statistically significant summary estimate of 1.01 per 1 kg increase in weight (95% CI: 1.00–1.01, *P*=0.001) with an *I*^*2*^ value of 0%. Meta-regressions of study design, country of study population, and data ascertainment method were all null.

**Figure 4:**
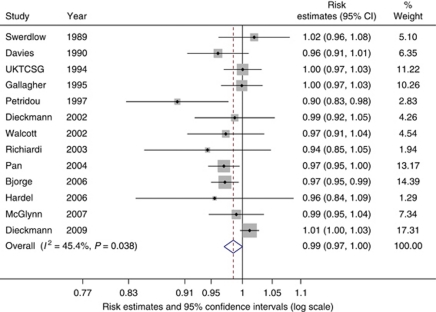
Forest plot of the association between body mass index (per kg m^−2^) and testicular cancer risk. Each study-specific estimate is represented by a small solid diamond with adjoining horizontal lines which represent the 95% confidence intervals. The size of the grey square surrounding the study-specific estimates represents the weight of each study in the meta-analysis. The diamond with an ascending dashed line from its upper point is the summary estimate. The width of diamond represents the 95% confidence intervals of the summary estimate.

BMI was analysed both as a continuous and as a categorical metric in relation to TGCT. Thirteen studies were included in the continuous meta-analysis in which we found a tentative inverse relationship with TGCT with a summary risk estimate of 0.99 per one unit increase in BMI (95% CI: 0.97–1.00, *P*=0.053; Figure 4). The *I*^*2*^ value was the lowest of all *I*^*2*^ values calculated for the continuous analyses at 45% (95% UI: 0–71%) indicating a moderate amount of between-study heterogeneity. The Egger and Begg's tests did not provide strong evidence for small-study effects (*P*=0.081 and *P*=0.393, respectively).

**Figure 5:**
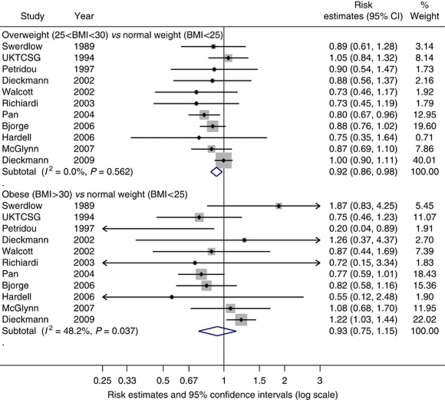
Forest plot of the association between body mass index (categorical) and testicular cancer risk. Each study-specific estimate is represented by a small solid diamond with adjoining horizontal lines which represent the 95% confidence intervals. The size of the grey square surrounding the study-specific estimates represents the weight of each study in the meta-analysis. The diamond with an ascending dashed line from its upper point is the summary estimate. The width of diamond represents the 95% confidence intervals of the summary estimate.

In total, 11 studies contributed to the categorical meta-analysis of BMI (kg m^−2^) and TGCT (Figure 5). For the categorical analyses, normal weight men (BMI <25) were used as the referent for comparison with overweight men (BMI 25–29.9), and obese men (BMI ⩾30). We found that overweight was inversely related to TGCT, compared with normal weight, with a summary OR of 0.92 per unit BMI (95% CI: 0.86–0.98, *P*=0.011) with no heterogeneity indicated (*I*^*2*^=0%). Sensitivity analysis demonstrated that the study by Dieckmann *et al* (2009) was particularly influential because of its large size, and thus high weighting in the BMI analysis. Moreover, its null finding is in contrast with that of most other studies. When this study was excluded from the meta-analysis, the summary estimate indicated an even stronger inverse relationship (OR=0.87 per unit BMI, 95% CI: 0.80–0.95, *I*^*2*^=7%). When we compared the obese group with the referent group, BMI was not related to TGCT, with a summary risk estimate of 0.92 per unit BMI (95% CI: 0.75–1.15, *P*=0.496) with an *I*^*2*^ of 46% (95% UI: 0–100%). Again, sensitivity analysis showed that the study by Dieckmann *et al* (2009) was highly influential and when this study was omitted, the obesity-TGCT meta-analysis indicated an association of borderline statistical significance (OR=0.85 per unit BMI, 95% CI: 0.71–1.01, *P*=0.068). The heterogeneity was lower after the omission of the Dieckmann *et al* (2009) study, but the uncertainty interval was just as large (*I*^*2*^=7%, 95% UI: 0–100%).

Variables assessed using meta-regression included country of study population (grouped by continent), study design, and data ascertainment method. Meta-regression of variables such as mean age at diagnosis and mean age of study participants could not be conducted because they were not available for all included studies.

## Discussion

In this systematic review and meta-analysis of body size in relation to TGCT, we found a positive association with height and an inverse association with BMI. The meta-analysis of weight and TGCT indicated no association. The association between height and testicular cancer has been examined by 17 studies, 13 of which were included in this meta-analysis. Although there was evidence of a moderate-to-high amount of heterogeneity among the 13 studies included, all but three reported a positive association (Swerdlow *et al*, 1989; Petridou *et al*, 1997; Hardell *et al*, 2006). Of the four studies (Brown *et al*, 1987; Thune and Lund, 1994; Davies *et al*, 1996; Rasmussen *et al*, 2003) that were not included in the meta-analysis because of missing or incomplete data, one (Davies *et al*, 1996) found a non-significant inverse association, whereas three (Brown *et al*, 1987; Thune and Lund, 1994; Rasmussen *et al*, 2003) found positive associations, although only one of these latter estimates was statistically significant at the 0.05 level (Rasmussen *et al*, 2003).

Both height and TGCT incidence follow a birth-cohort effect (Bergstrom *et al*, 1996; Cole, 2000; McGlynn *et al*, 2003) and are thought to be influenced by a combination of genetic (Biermann *et al*, 2007), perinatal (Ekbom, 1998), and early childhood factors (Meyer and Selmer, 1999; Bjorge *et al*, 2006). There is some evidence to suggest that testicular cancer is associated with increased consumption of calories (Sigurdson *et al*, 1999), fat (Armstrong and Doll, 1975; Paulozzi, 1998; Sigurdson *et al*, 1999), and dairy products (1971; Armstrong and Doll, 1975; Davies *et al*, 1996; Ganmaa *et al*, 2002; Stang *et al*, 2006) during the developmental period, although the literature is not entirely consistent (Bonner *et al*, 2002; Walcott *et al*, 2002; McGlynn *et al*, 2007), which is likely the result of the low sensitivity and misclassification associated with retrospective ascertainment of exposure over a long time period. However, these consumption hypotheses could conceivably be explained by the birth-cohort effects, including the declined incidence of testicular cancer observed in certain western European countries during the Second World War (Moller, 1989), and the subsequent increased incidence thereafter (Chia *et al*, 2010). Alternatively, height could be a proxy for testis size, given evidence that these anthropometric features are correlated (Handelsman and Staraj, 1985; Sobowale and Akiwumi, 1989), and an increased testis size may relate to an increased testicular cancer risk. Arguing against this hypothesis is a study of 1700 newborn boys that found testis size was larger in Finnish than Danish neonates, (Main *et al*, 2006), although testicular cancer rates are much higher in Denmark than in Finland (Chia *et al*, 2010). Another hypothesis is that cryptorchidism may be associated with height, although Taskinen and Wikstrom (2004) found no evidence of this association. In addition, we find no evidence for such a relationship in our US Servicemen's Testicular Tumor Environmental and Endocrine Determinants (STEED) case-control study (McGlynn *et al*, 2007; unpublished data), which is also supported by the findings of a previous study (Schnakenburg *et al*, 1977). Other studies aiming to elucidate the association between height and TGCT have assessed insulin-like growth factor and insulin-like growth factor-binding proteins (Juul *et al*, 1994; Khandwala *et al*, 2000; Chia *et al*, 2008), and early onset of puberty (Hagg and Taranger, 1992; Karlberg, 2002), but results have predominantly supported the null hypothesis.

We found no evidence of an association between weight and TGCT. The studies examined reported a slightly positive to slightly negative associations, but none of the ORs attained statistical significance. Four studies could not be included because data were unavailable or authors could not be contacted (Lin and Kessler, 1979; Whittemore *et al*, 1984; Davies *et al*, 1996; Rasmussen *et al*, 2003). Weight adjusted for height, as in the BMI metric, is likely to be a more sensitive marker of excess body fat providing increased statistical power to detect any association with TGCT.

The summary OR of both the continuous and categorical analyses implied an inverse association of BMI with TGCT risk. Of the four studies (Lin and Kessler, 1979; Kleinteich and Marx, 1983; Thune and Lund, 1994; Rasmussen *et al*, 2003) not included in the BMI analyses, two found a positive association between BMI and TGCT (Lin and Kessler, 1979; Thune and Lund, 1994), one found a trend toward an inverse association (Kleinteich and Mark, 1983), and one found no association (Rasmussen *et al*, 2003). It is possible that the inclusion of one or more of these studies in our meta-analyses could have influenced the BMI meta-analyses. If the two excluded studies reporting a positive association (Lin and Kessler, 1979; Thune and Lund, 1994) had been included, the overall effect estimate would have been weakened (Lin and Kessler, 1979; Thune and Lund, 1994). Of the studies that were included, the study by Dieckmann *et al* (2009) was highly influential, especially in the categorical analyses; when it was removed from the overweight against normal weight categorical BMI analysis, the summary estimate remained statistically significant and the confidence interval tightened (OR=0.87, 95% CI: 0.80, 0.95). When the Dieckmann (2009) study was removed from the obese against normal weight categorical BMI analysis, the summary estimate did not reach statistical significance (OR=0.85, 95% CI: 0.71, 1.01). However the heterogeneity (*I*^*2*^) decreased significantly, from 48% to 7%. This study did differ somewhat in terms of study design when compared with the other studies included in the categorical BMI meta-analyses. It was a large, multi-centre case-control study in which cases were enroled both prospectively and retrospectively during a specified period. Controls were taken from the German National Health Survey; because TGCTs are more prevalent in younger men, only men aged 18–49 years were abstracted and chosen for these analyses. The authors could not calculate relative risks because the exact BMI of control individuals was not known; rather, BMI data was ascertained in categories of exposure (Dieckmann *et al*, 2009). Despite the differences, there is little reason to believe that they would contribute to a false-positive finding. The large study size, including nearly 8500 cases, gives a cause to believe that the findings in this study may be equally, if not more, valid relative to other studies.

The current study had several notable strengths. The search strategy was extremely thorough: we used a very broad search and narrowed our findings by manually reviewing titles, abstracts, and articles. In addition, we did not exclude studies based on language or year of report, and we systematically contacted authors in requests for additional information/data. Unadjusted estimates for OR were included in these meta-analyses; thus all the statistical models were equivalent. This may be a preferred strategy as opposed to combining estimates adjusted for different sets of variables. In addition, height does not vary greatly by age in adulthood, so age as a potential confounder would only pertain to the BMI and weight meta-analyses. We also checked for any evidence of small-study biases by examining funnel plots with Egger and Begg's tests, as well as sensitivity analyses to assess the impact of each individual study on summary estimates. In addition, this is the first meta-analysis of the variables height (continuous), weight (continuous), and BMI (continuous and categorical) in relation to TGCT risk.

This analysis also has several limitations. It was not feasible to include every identified study in the meta-analysis, because three cohort studies (Whittemore *et al*, 1984; Thune and Lund, 1994; Rasmussen *et al*, 2003) did not provide the necessary statistics for their inclusion and the methods employed do not lend themselves to estimation of hazard ratios. In addition, five case-control studies had collected the relevant data but did not provide the appropriate ORs, and unfortunately this data could not be obtained through correspondence (Lin and Kessler, 1979; Depue *et al*, 1983; Kleinteich and Marx, 1983; Brown *et al*, 1987; Davies *et al*, 1996). However, these eight studies were included in the discussion of the results we present herein. A second limitation is that the estimates used for the meta-analyses were unadjusted, and thus potential confounding variables could not be taken into account. However, as previously mentioned, by calculating the individual study estimates using the same statistical method, we were able to increase the validity of our meta-analytic approach. Also, there is very little difference between unadjusted/minimally adjusted and adjusted estimates in studies which have published such (UKTCSG, 1994; Gallagher *et al*, 1995; Dieckmann and Pichlmeier, 2002; Richiardi *et al*, 2003; McGlynn *et al*, 2007). Furthermore, given that the mechanism of the relationship of body size and TGCT is not well understood, and that no variable has been shown to consistently and significantly alter such, we believe the most prudent approach is to combine unadjusted study-specific estimates rather than pursue a strategy based on artificial adjustment. A third limitation is that we could not identify the cause(s) of heterogeneity detected in the meta-analysis of height and TGCT even though we employed meta-regression using variables specified *a priori*. The high levels of heterogeneity do not invalidate the findings presented herein, but they do warrant somewhat of a cautious interpretation. A fourth limitation is that weight was not measured in a uniform manner throughout the included studies. Rather, weight was measured at different points before or at the time of diagnosis, as specified in the individual study methods and summarised above. Weight can vary greatly throughout a person's life, and it may have more or less influence at different points in the disease process. Thus, to ensure meta-analytic validity and minimise heterogeneity, it is important that weight is collected at similar points in the disease process for each study included. The studies included here were limited to those in which weight was measured at or before diagnosis; post-diagnostic weight is more likely to be a reflection of treatment and stress associated with disease. Differences in the timing and method of weight measurement could have contributed to the heterogeneity of the weight meta-analyses, although a sensitivity analysis of the included studies demonstrated that no single study was dominant. One final limitation is the lack of prospective studies available for inclusion in this meta-analysis. There are very few cohort studies looking at the relationship between body size and TGCTs, and only one could be included in these meta-analyses (Bjorge *et al*, 2006). Given the rarity of TGCT, cohort studies typically accrue few cases, making this an inefficient study design when one considers the balance of statistical power to financial costs. The findings of Bjorge *et al* (2006) were comparable to the overall summary estimates from our meta-analyses; there was a small positive relationship between height and TGCTs, a small inverse relationship between BMI and TGCTs, and no association between weight and TGCTs.

This study provides support for a positive association between height and TGCT, but little support for an association between weight and TGCT. Further investigation of the inverse relationship between BMI and TGCT may be warranted, for which the present findings lend only limited support.

## Figures and Tables

**Table 1:** Studies included in the meta-analytic models

**Title**	**First author**	**Year**	**Country**	**Number of cases**	**Study design**	**Ascertainment method**	**Risk estimate: height**	**Risk estimate: weight**	**Risk estimate: BMI**
Testis cancer: post-natal hormonal factors, sexual behaviour and fertility (Swerdlow *et al*, 1989)	Swerdlow	1989	UK	259	Case-control	Self-report	0.96 (0.85, 1.07)	—	1.02 (0.96, 1.08)
Body size and cancer of the testis (Davies *et al*, 1990)	Davies	1990	Denmark	438	Case-control	Record/registry	1.01 (0.93, 1.10)	0.99 (0.98, 1.00)	0.96 (0.91, 1.01)
Social, behavioural and medical actors in the aetiology of testicular cancer: results from the UK study (UKTCSG, 1994)	UKTCSG	1994	UK	794	Case-control	Self-report	1.05 (0.97, 1.14)	1.00 (0.99, 1.01)	1.00 (0.97, 1.03)
Physical-activity, medical history, and risk of testicular cancer (Alberta and British-Columbia, Canada) (Gallagher *et al*, 1995)	Gallagher	1995	Canada	510	Case-control	Self-report	1.20 (1.09, 1.33)	1.01 (1.00, 1.02)	1.00 (0.97, 1.03)
Baldness and other correlates of sex hormones in relation to testicular cancer (Petridou *et al*, 1997)	Petridou	1997	Greece	97	Case-control	Self-report	0.98 (0.82, 1.18)	0.97 (0.95, 0.99)	0.90 (0.83, 0.98)
Is risk of testicular cancer related to body size? (Dieckmann and Pichlmeier, 2002)	Dieckmann	2002	Germany	353	Case-control	Self-Report	1.35 (1.20, 1.52)	1.02 (0.99, 1.04)	0.99 (0.92, 1.05)
A case-control study of dietary phytoestrogens and testicular cancer risk (Walcott *et al*, 2002)	Walcott	2002	US	159	Case-control	Self-report	1.15 (0.98, 1.35)	1.00 (0.98, 1.01)	0.97 (0.91, 1.04)
Body size at birth and adulthood and the risk for germ-cell testicular cancer (Richiardi *et al*, 2003)	Richiardi	2003	Sweden	371	Case-control	Record/registry	1.17 (1.05, 1.30)	1.01 (0.99, 1.03)	0.94 (0.85, 1.05)
Association of obesity and cancer risk in Canada (Pan *et al*, 2004)	Pan	2004	Canada	685	Case-control	Self-report	1.26 (1.19, 1.34)	1.01 (1.00, 1.02)	0.97 (0.95, 1.00)
*In utero* exposure to persistent organic pollutants in relation to testicualr cancer risk (Hardell *et al*, 2006)	Hardell	2006	Sweden	58	Case-control	Self-report	0.96 (0.68, 1.36)	0.97 (0.94, 1.00)	0.96 (0.84, 1.09)
The impact of height and body mass index on the risk of testicular cancer in 600 000 Norwegian men (Bjorge *et al*, 2006)	Bjorge	2006	Norway	1004	Cohort	Record/registry	1.08 (1.03, 1.13)	1.00 (0.99, 1.01)	0.97 (0.95, 0.99)
Adolescent milk fat and galactose consumption and testicular germ cell cancer (Stang *et al*, 2006)	Stang	2006	Germany	269	Case-control	Self-report	1.23 (1.11, 1.37)	1.01 (0.99, 1.02)	—
Body size, dairy consumption, puberty, and risk of testicular germ cell tumors (McGlynn *et al*, 2007)	McGlynn	2007	US	767	Case-control	Self-report	1.16 (1.08, 1.25)	1.01 (1.00, 1.02)	0.99 (0.95, 1.04)
Is increased body mass index associated with the incidence of testicular germ cell cancer? (Dieckmann *et al*, 2009)	Dieckmann	2009	Germany	8498	Case-control	Self-report	—	—	1.01 (1.00, 1.03)

Abbreviations: BMI=body mass index; UK=United Kingdom; US=United States.
